# Support needs of Dutch young adult childhood cancer survivors

**DOI:** 10.1007/s00520-021-06723-7

**Published:** 2022-01-04

**Authors:** L. M. E. van Erp, H. Maurice-Stam, L. C. M. Kremer, W. J. E. Tissing, H. J. H. van der Pal, L. Beek, A. C. H. de Vries, M. M. van den Heuvel-Eibrink, B. A. B. Versluys, M. van der Heiden-van der Loo, M. van Gorp, G. A. Huizinga, M. A. Grootenhuis

**Affiliations:** 1 Princess Máxima Center for Pediatric Oncology, Heidelberglaan 25, 3584CS Utrecht, The Netherlands; 2 Emma Children’s Hospital, Amsterdam UMC, University of Amsterdam, Locatie AMC Meibergdreef 9, 1105AZ Amsterdam, The Netherlands; 3 Beatrix Children’s Hospital/University of Groningen/University Medical Center Groningen, Hanzeplein 1, 9713 GZ Groningen, The Netherlands; 4 Sophia Children’s Hospital/Erasmus Medical Center, Wytemaweg 80, 3015CN Rotterdam, The Netherlands; 5 Wilhelmina Children’s Hospital/University Medical Center Utrecht, Lundlaan 6, 3584 EA Utrecht, The Netherlands; 6 Dutch Childhood Oncology Group, Heidelberglaan 25, 3584CS Utrecht, The Netherlands

**Keywords:** Childhood cancer, Young adults, Survivorship care, Psychosocial support, Needs

## Abstract

**Background:**

Studies about support needs of young adult childhood cancer survivors (YACCS) previously focused mainly on information needs. This study assessed support needs and associated factors (sociodemographic, medical, and psychosocial functioning) in Dutch YACCS.

**Methods:**

YACCS (aged 18–30, diagnosed ≤ 18 years, time since diagnosis ≥ 5 years) cross-sectionally filled out a questionnaire regarding their need for various types of support (concrete information, personal counseling, and peer contact) in eight domains (physical consequences of childhood cancer, social-emotional consequences, relationships and sexuality, fertility, lifestyle, school and work, future perspective, insurance and mortgage), and questionnaires assessing health-related quality of life (PedsQL-YA), anxiety and depression (HADS), and fatigue (CIS-20R). Descriptive statistics were used to describe support needs. Linear regression was used to identify characteristics associated with support needs.

**Results:**

One hundred fifty-one YACCS participated (response = 40%). Most YACCS reported a need for support in one or more domains (88.0%, *N* = 133). More than half of the participants reported a need for concrete information in the domains lifestyle, fertility, and physical consequences of childhood cancer and 25–50% in the domains insurance and mortgages, future perspective, and social-emotional consequences of childhood cancer. In the domains lifestyle and physical as well as emotional consequences of childhood cancer, 25–50% reported a need for counseling. Overall need for support was positively associated with middle (β = 0.26, *p* = 0.024) and high (β = 0.35, *p* = 0.014) compared to low educational attainment and (sub)clinical anxiety (β = 0.22, *p* = 0.017), and negatively associated with social functioning (β =  − 0.37, *p* = 0.002) in multivariate analyses.

**Conclusion:**

YACCS report the strongest need for support, for concrete information, in the domains lifestyle, fertility, and physical consequences of childhood cancer. Associated factors were mostly socioeconomic and psychosocial in nature. Psychosocial care should be an integral part of survivorship care for YACCS, with screening for psychosocial problems, information provision including associated emotional consequences and support if necessary (psycho-education) and tailored interventions, and adequate referrals to more specialized care if necessary.

## Introduction

In 2020, the number of childhood cancer survivors (CCS) in Europe reached 500,000 [1, 2]. Due to childhood cancer treatment, many CCS experience late effects, (chronic) health problems that may manifest up until many years after the end of treatment [3, 4]. Besides physical late effects, CCS may experience psychosocial problems and impaired quality of life [5, 6]. Therefore, survivorship care aiming at both their physical and psychosocial health is crucial in keeping CCS as healthy as possible after treatment. Current standards of care recommend that survivorship care should contain routine screening and provision of psychosocial interventions in order to optimize early detection and treatment of psychosocial problems [7]. However, limited data is available about what CCS themselves report to need in terms of psychosocial support during survivorship care.

Previous studies on needs in adult CCS and survivors of adolescent and young adult (AYA) cancer focused on need for information, showing that these populations reported unmet needs, especially information regarding their illness, late effects, lifestyle, and sexual issues [8–13]. Unmet information needs in CCS and AYA cancer survivors were found to be associated with psychosocial problems such as anxiety, depression, distress, and a lower quality of life [10, 11, 14]. Furthermore, unmet information needs can negatively impact survivorship care attendance [10, 12]. Knowing the needs of CCS could help tailor the content of psychosocial survivorship care to the needs of CCS, which may foster engagement with survivorship care in this population.

Psychosocial support during survivorship care can include psycho-education (concrete information, associated emotional consequences and support aimed at improving coping and self-management) about the diagnosis, treatment and late effects, counseling (psychological interventions or therapy), and peer contact (e.g., group meetings). A few studies have explored needs in a broader context than information needs. One large study found needs related to psycho-emotional problems, coping, care, and support as well as a need for cancer- and treatment-related information in CCS [15]. A recent qualitative study from Switzerland also provided insight into the needs of adult CCS beyond need for information, showing that survivors have unmet needs for psychosocial support [16].

Insight in the needs of young adult CCS in survivorship care (YACCS, 18–30 years old) may be especially impactful to long-term health and well-being of CCS. Young adulthood is an important developmental stage with many challenges. This life phase is marked by the development of autonomy and identity [17]. The experience of childhood cancer and late effects was found to hinder YACCS’ development in terms of achieved milestones regarding autonomy development, psycho-sexual development, and social development [18]. This delay in development may influence their quality of life [19]. Thus, young adulthood may be the prime time to empower YACCS to take control of their own health. In addition, various studies have shown that YACCS are vulnerable to psychosocial problems, such as reduced (health-related) quality of life and higher levels of distress, anxiety, depression, post-traumatic stress disorder (PTSD), and fatigue [5, 6, 20–24]. One of our recent studies on Dutch YACCS showed that their psychosocial well-being is worse than that of Dutch peers, and that the impact of cancer played an important role in explaining psychosocial well-being [25]. Therefore, YACCS could benefit from psychosocial surveillance and support as a part of their survivorship care.

Insight in the needs of YACCS may improve the attendance of survivorship care of this vulnerable population in the middle of crucial development, so their psychosocial well-being can be surveilled and supported. However, evidence on the specific needs of YACCS is scarce. YACCS are often researched in combination with adolescent and young adult cancer patients, or survivors of cancer during young adulthood. A qualitative study found that YACCS and survivors of AYA cancer describe similar resource needs: age-appropriate information, peer support, and proactive attention for salient issues by health care professionals [26]. Besides common challenges (physical appearance, fertility late effects, social relationships, and changing priorities), difficulty with identity formation, social isolation, and complex health care transitions were identified as issues specifically important to YACCS [26].

Insight into the needs of YACCS can be used to tailor psychosocial support during survivorship care to YACCS needs. Therefore, the aims of the present study are to assess Dutch YACCS’ support needs in various domains and to examine whether need for support is associated with sociodemographic and medical characteristics of YACCS as well as with their psychosocial well-being.

## Methods

The Dutch LAnge TERmijn (LATER, translation: long term) registry contained 946 eligible YACCS, aged 18–30, diagnosed at age 0–18, ≥ 5 years since diagnosis, and treated at one of four participating Dutch pediatric oncology centers (located in Amsterdam, Rotterdam, Utrecht, and Groningen). A total of 400 YACCS were randomly selected by a data manager from the pseudonymized Dutch LATER registry. The selection was stratified in order to have an equal representation of men and women, and of age groups (18–25 and 26–30 years) and diagnosis age groups (0–7, 7–13, and 13–18 years) to account for differences in developmental stage.

After excluding 22 YACCS who had no known address, were living abroad, or were recently deceased, 378 eligible YACCS were invited by the researchers with an information letter in the mail in June of 2018. YACCS could fill out questionnaires on paper or online. Participants provided written informed consent and the Medical Ethical Committee of the University Hospital Utrecht reviewed this study (case number 18/256). Patient information letters were presented to members of the survivor committee of the Dutch Childhood Cancer Association in order to assure appropriate use of understandable language.

### Measures

#### Sociodemographic characteristics

In a short list of sociodemographic questions, date of birth, sex, partner status, number of children, employment status, and attained and current education (low = primary education, lower vocational education, lower and middle general secondary education; middle = middle vocational education, higher general secondary education, pre-university education; high = higher vocational education, university) were asked.

#### Medical characteristics

The Dutch LATER registry provided data on the initial cancer diagnosis and treatment as well as recurrences and aggregated data about non-participants.

#### Need for support

Support needs were assessed using a questionnaire made specifically for the purpose of this study focusing on different domains of support and types of support, based on literature and clinical experience of hospital psychologists and survivorship care doctors (Appendix A). YACCS were asked to indicate need for support in the following eight predefined domains: physical consequences of childhood cancer, social and emotional consequences of childhood cancer, relationships and/or sexuality, fertility, lifestyle and health risks after childhood cancer, choices relating to school and work, future perspective, and insurance and mortgages. YACCS could also indicate any other areas where they need support. For each domain, YACCS could indicate whether they felt a need for one or multiple support types by ticking one or multiple boxes: concrete information, personal counseling, peer support, other support, or no support needed. A total needs score was calculated as a sum score (range: 0–9) indicating in how many domains YACCS reported need for at least one support type.

#### Health-related quality of life (HRQOL)

The Pediatric Quality of Life Inventory Young Adults (PedsQL-YA) measures generic HRQOL. The PedsQL-YA has four scales (Physical, Emotional, Social, and Work/School Functioning), a total scale and a Psychosocial Summary Scale combining emotional, social, and work/school functioning. Higher scores (range 0–100) represent better HRQOL. The PedsQL-YA has good psychometric properties and a reference group of Dutch young adults is available [27]. This study made use of the scales Physical, Social, and Work/School Functioning. The Emotional Functioning scale, total scale, and Psychosocial Summary Scale of the PedsQL-YA were not used because of correlation with the scores on the anxiety and depression measurement.

#### Anxiety and depression

The Hospital Anxiety and Depression Scale (HADS) aims to measure levels of anxiety and depression in separate scales [28]. Scale scores ≥ 8 for anxiety and depression are considered (sub)clinical. The HADS has good psychometric properties [29] and a reference group of Dutch young adults is available [30].

#### Fatigue

The Checklist Individual Strength Revised (CIS-20R) measures fatigue, and consists of four scales: Fatigue Severity, Concentration, Motivation, and Activity [31]. Higher scores indicate higher levels of fatigue and fatigue-related impairment. Fatigue severity was used in the current study, with a score of 35 or more classified as severe fatigue [31]. The CIS-20R has good psychometric properties and a reference group of Dutch young adults is available [31].

### Statistics

Statistical tests were performed using IBM SPSS version 25. All tests were two-sided. Before conducting the main analyses, several preparatory analyses were conducted. First, missing data were imputed on the basis of the guidelines of the questionnaires used. Second, the internal consistency of each scale used in the analyses was calculated, yielding satisfactory Cronbach’s α: PedsQL-YA 0.80 ≤ α ≤ 0.84; HADS 0.79 ≤ α ≤ 0.88; CIS-20R fatigue severity scale α = 0.78.

Differences between participants and non-participants on available sociodemographic and medical characteristics were tested using one-sample *t* tests and binominal tests.

To characterize the sample, psychosocial functioning of the YACCS, as measured with the PedsQL-YA, HADS, and CIS-20R, was compared to reference groups of Dutch young adults with ANOVA or logistic regression, as reported in a previous study [25].

To answer our first research question about the assessment of YACCS’ support needs, an overview of support needs was created by calculating frequencies for each support type per domain. Then, to describe need for support, two scores were calculated: (1) a dichotomous domain score indicating whether or not a YACCS reported need for at least one support type in a domain and (2) a needs sum score (range: 0–9) indicating in how many domains YACCS reported need for at least one support type.

To study associations of support needs with sociodemographic (sex, attained education, partner status), medical (age at diagnosis, time since diagnosis, diagnosis, treatment, recurrence) characteristics as well as psychosocial outcomes (PedsQL-YA physical and social functioning scales, dichotomous HADS anxiety ≥ 8, dichotomous HADS depression ≥ 8, dichotomous CIS-20R fatigue severity ≥ 35), multivariate linear regression analysis for the needs sum score was performed with the aforementioned characteristics as independent variables. To gain more detailed insight, separate multivariate logistic regression analyses were performed exploratively for each of the eight dichotomous domain scores. To reduce the number of independent variables in the multivariate logistic regression analyses, independent variables were selected if they were univariately associated with the dichotomous domain score at α = 0.05. For each dichotomous domain score, the selected independent variables were entered into the multivariate models at once (Table 3).

## Results

### Sample characteristics

A total of 151 YACCS (61.6% female, mean age 24.1 SD 3.6, mean time since diagnosis 13.6 SD3.8) participated by returning a completed questionnaire (response rate = 40%). Participants were significantly more often female (*p* ≤ 0.001) and had less often received a bone marrow transplantation (BMT) (*p* = 0.012) than non-participants (Table 1). YACCS’ scores on the HRQOL scales were lower than those of the general population, and YACCS were more likely to experience anxiety and severe fatigue than the general population (study reported elsewhere [25]).

**Table 1:** Characteristics of participants and non-participants

	Participants (*N* ≈ 151)^a^	Non-participants (*N* = 223)^b^	*p*
**Sociodemographic characteristics**			
*Age (years, mean ± SD (range))*	24.1 ± 3.6 (18–30)	24.0 ± 3.4 (18–30)	0.659
*Sex (female*, *N*(%))	61.6 (93)	40.8 (90)	≤ 0.001
*Partner status* *N*(%)			
Yes	51.0 (75)		
No	49.0 (72)		
*Employment status N*(%)			
Paid occupation	70.9 (105)		
Without paid occupation	29.1 (43)		
*Attained education*^c^ *N*(%)			
Low	19.3 (28)		
Middle	48.3 (70)		
High	32.4 (47)		
*Current education*^c^ *N*(%)			
Low	3.1 (2)		
Middle	27.7 (18)		
High	69.2 (45)		
**Medical characteristics**			
*Age at diagnosis (years, mean ± SD (range))*	10.5 ± 4.5 (0.4–17)	10.6 ± 4.5 (0–18)	0.756
*Time since diagnosis (years, mean ± SD (range))*	13.6 ± 3.8 (6–27)	13.5 ± 3.7 (6–28)	0.652
*Diagnosis N*(%)			
Hematologic cancers	66.9 (101)	61.7 (142)	0.119
CNS tumors	8.6 (13)	9.9 (22)	0.358
Solid tumors	24.5 (37)	28.3 (63)	0.173
Recurrence *N*(%)	13.9 (21)		
Treatment^d^ *N*(%)			
Surgery (S)	61.6 (93)	63.7 (142)	0.323
Chemotherapy (CT)	95.4 (144)	95.5 (213)	0.522
Radiotherapy (RT)	37.1 (56)	35.0 (78)	0.323
SCT/BMT	7.3 (11)	13.5 (30)	0.012
*Treatment combinations*^e^ N(%)			
CT only	32.5 (49)		
CT + RT	6.0 (9)		
RT + S	4.6 (7)		
CT + S	30.5 (46)		
CT + S + RT	26.5 (40)		
	Participants (*N* ≈ 151)	General population^f,g^	*p *
**Psychosocial well-being** mean ± SD (range) / % (N)			
*PedsQL-YA physical*	80.2 ± 19.7 (21.9–100)	87.1 ± 16.0	≤ 0.001
*PedsQL-YA social*	82.1 ± 20.0 (10.0–100)	87.2 ± 14.5	0.001
*PedsQL-YA school/work*	76.8 ± 19.1 (5.0–100)	82.3 ± 15.7	≤ 0.001
*HADS anxiety (≥ 8)*	30.2 (45)	18.8 (42)	0.017
*HADS depression (≥ 8)*	12.8 (19)	7.6 (17)	0.134
*CIS-20R fatigue severity (≥ 35)*	36.2 (54)	20.8 (55)	≤ 0.001

^a^Data incomplete for some participants. The numbers in the table are based on the records with complete data per variable

^b^No medical information available from 4 non-participants

^c^Low = primary education, lower vocational education, lower and middle general secondary education; middle = middle vocational education, higher general secondary education, pre-university education; high = higher vocational education, university

^d^More than one category possible

^e^Treatments for primary tumor and (if applicable) recurrence(s)

^f^PedsQL-YA *N* = 649; HADS *N* = 224; CIS-20-R *N* = 264

^g^Study reported elsewhere[25]

Abbreviations: *PedsQL-YA* Pediatric Quality of Life–Young Adults; *HADS* Hospital Anxiety and Depression Scale; *CIS-20R* Checklist Individual Strength Revised

### Support needs

Most YACCS reported a need for support in one or more domains (88.0%). On average, YACCS reported any need of support in 4.4 domains (SD = 2.6, range = 0–9). The percentage of YACCS reporting any need for support in the various domains was 76.2% for lifestyle and health risks after childhood cancer, 69.5% for physical consequences of childhood cancer, 68.2% for fertility, 54.3% on insurances and mortgages, 53.6% for social-emotional consequences of childhood cancer, 49.0% on future perspective, 34.4% for relationships and sexuality, 29.8% on school and work, and 4.6% on other domains.

**Fig. 1:**
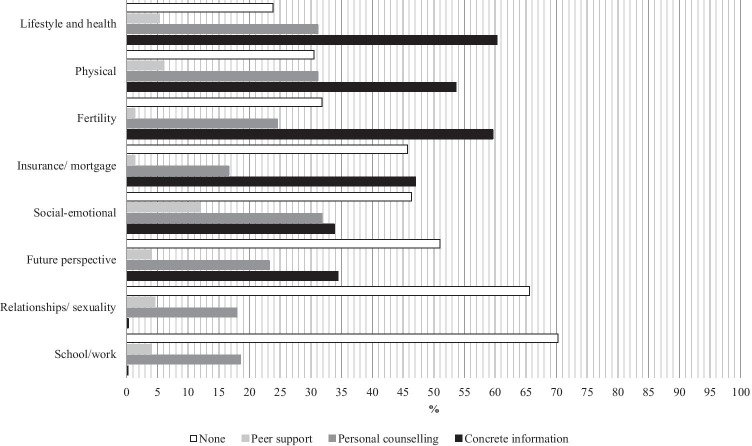
Needs of YACCS in eight domains by support type

Figure 1 shows the percentage of YACCS indicating a need for information, counseling, and peer contact in each domain. On all domains except for school and work, concrete information was the support type most mentioned. More than half of the participating YACCS reported a need for concrete information in the domains lifestyle and health risks after childhood cancer, fertility, and physical consequences of childhood cancer. Between 25 and 50% of YACCS reported a need for concrete information about insurances or mortgages, future perspective, and social-emotional consequences of childhood cancer. Also 25 to 50% of YACCS reported a need for personal counseling on lifestyle and health, and physical as well as social-emotional consequences of childhood cancer. Need for peer support was reported in all domains ranging from 1.3 in fertility and insurance/mortgage to 11.9% in social-emotional consequences. Very few YACCS reported a need for types of support other than concrete information, personal counseling, or peer support, so the corresponding percentages were not shown in the figure.

### Associations between need for support and sociodemographic and medical characteristics as well as psychosocial-well-being

In multivariate linear regression analysis, the needs sum score was significantly positively associated with middle (β = 0.26, *p* = 0.024) and high (β = 0.35, *p* = 0.014) compared to low educational attainment, as well as with (sub)clinical anxiety (β = 0.22, *p* = 0.017), and negatively associated with social functioning (β =  − 0.37, *p* = 0.002). The full model explained 58.7% of variance in needs (Table 2).

**Table 2:** Multivariate linear regression model for support needs with sociodemographic and medical characteristics as well as psychosocial well-being as independent variables; *N* = 143^a^

	**Total needs score**				
	*β*	*B*	*95%CI*	*p*	
**Sociodemographic**					
*Sex (ref* = *male)*	0.14	0.74	[− 0.09;1.57]	0.080	
*Attained education (ref* = *low)*					
Middle	0.26	1.29	[0.18;2.41]	0.024	
High	0.35	1.85	[0.39;3.31]	0.014	
**Medical**					
*Age at diagnosis*	− 0.06	− 0.03	[− 0.17;0.11]		0.652
*Time since diagnosis*	− 0.10	− 0.07	[− 0.21;0.08]		0.359
*Diagnosis (ref* = *hematological)*					
CNS tumor	− 0.12	− 1.15	[− 3.20;90]		0.268
Solid tumor	− 0.001	0.01	[− 1.12;1.11]		0.991
*Recurrence*	0.12	0.86	[− 0.31;2.02]		0.147
*Surgery (yes/no)*	− 0.03	− 0.17	[− 1.18;0.84]		0.740
*Chemotherapy (yes/no)*	− 0.18	− 2.04	[− 4.15;0.08]		0.059
*Radiotherapy (yes/no)*	− 0.09	− 0.47	[− 1.46;0.52]		0.352
**Psychosocial**					
*PedsQL-YA Physical Functioning*	0.03	0.003	[− 0.02;0.03]	0.820	
*PedsQL-YA Social Functioning*	− 0.37	− 0.05	[− 0.08; − 0.02]	0.002	
*PedsQL-YA Work/School Functioning*	− 0.05	− 0.01	[− 0.03;0.02]	0.653	
*HADS (sub)clinical anxiety (*≥ *8)*	0.22	1.22	[0.22;2.21]	0.017	
*HADS (sub)clinical depression (*≥ *8)*	− 0.10	− 0.77	[− 2.14;0.61]	0.273	
*CIS-20R severe fatigue (*≥ *35)*	0.09	0.47	[− 0.46;1.40]	0.316	
*R*^2^	0.587				

^a^Number of respondents who completed all questionnaires

Abbreviations: *CNS* central nervous system; *PedsQL-YA* Pediatric Quality of Life–Young Adults; *HADS* Hospital Anxiety and Depression Scale; *CIS-20R* Checklist Individual Strength Revised

Need for support in the various domains was explored in more detail using multivariate logistic regression analyses, the results of which are displayed in Table 3.

**Table 3:** Multivariate logistic regression models for any support needs on the eight domains with sociodemographic and medical characteristics as well as psychosocial well-being as independent variables

	**Physical**				**Social-emotional**				**Relationships/sexuality**					**Fertility**			
	(69.5%, *N* = 105)^a^				(53.6%, *N* = 81) ^a^				(34.4%, *N* = 52) ^a^					(68.2%, *N* = 103) ^a^			
	OR	95%CI		*p*	OR	95%CI		*p*	OR	95%CI			*p*	OR	95%CI	*p*	
**Socioeconomic**																	
*Sex (ref* = *male)*	1.58	[0.73;3.42]		0.249	-	-		-	1.74	[0.76;3.98]			0.189	4.26	[1.94;9.38]	≤ 0.001	
*Attained Education (ref* = *low)*																	
Middle	-	-		-	-	-		-	-	-			-	2.42	[0.93;6.28]	0.070	
High	-	-		-	-	-		-	-	-			-	8.43	[2.55;27.82]	≤ 0.001	
**Medical**																	
*Age at diagnosis*	-	-		-	-	-		-	-	-			-	-	-	-	
*Time since diagnosis*	-	-		-	-	-		-	-	-			-	-	-	-	
*Diagnosis (ref* = *hematological)*																	
CNS tumor	-	-		-	-	-		-	-	-			-	-	-	-	
Solid tumor	-	-		-	-	-		-	-	-			-	-	-	-	
*Recurrence*	-	-		-	-	-		-	2.62	[0.94;7.32]			0.067	-	-	-	
*Surgery (yes/no)*	-	-		-	-	-		-	-	-			-	-	-	-	
*Chemotherapy (yes/no)*	-	-		-	-	-		-	-	-			-	-	-	-	
*Radiotherapy (yes/no)*	-	-		-	-	-		-	-	-			-	-	-	-	
**Psychosocial**																	
*PedsQL-YA Physical Functioning (0–100)*	-	-		-	1.03	[1.0;1.06]		0.065	-	-			-	-	-	-	
*PedsQL-YA Social Functioning (0–100)*	0.98	[0.96;1.01]		0.135	0.91	[0.88;0.95]		≤ 0.001	0.98	[0.96;1.0]			0.021	-	-	-	
*PedsQL-YA Work/School Functioning (0–100)*	-	-		-	0.99	[0.96;1.02]		0.574	-	-			-	-	-	-	
*HADS (sub)clinical anxiety (*≥ *8)*	4.29	[1.36;13.53]		0.013	5.66	[2.05;15.65]		0.001	2.24	[0.92;5.44]			0.075	-	-	-	
*HADS (sub)clinical depression (*≥ *8)*	-	-		-	0.44	[0.08;2.26]		0.322	1.04	[0.30;3.64]			0.954	-	-	-	
*CIS-20R severe fatigue (*≥ *35)*	2.11	[0.81;5.50]		0.126	-	-		-	-	-			-	-	-	-	
	**Lifestyle and health**				**School and work**				**Future perspective**					**Insurance and mortgage**			
	(76.2%, *N* = 115) ^a^				(29.8%, *N* = 45) ^a^				(49.0%, *N* = 74) ^a^					(54.3%, *N* = 82) ^a^			
	OR	95%CI	*p*		OR		95%CI	*p*	OR		95%CI	*p*		OR	95%CI		*p*
**Socioeconomic**																	
*Sex (ref* = *male)*	2.39	[1.01;5.66]	0.047		-		-	-	1.96		[0.86;4.49]	0.110		1.76	[0.81;3.84]		0.152
*Attained education (ref* = *low)*																	
Middle	2.14	[0.77;5.93]	0.146		-		-	-	-		-	-		-	-		-
High	6.90	[1.89;25.19]	0.003		-		-	-	-		-	-		-	-		-
**Medical**																	
*Age at diagnosis*	-	-	-		-		-	-	-		-	-		1.10	[1.02;1.20]		0.019
*Time since diagnosis*	-	-	-		-		-	-	-		-	-		-	-		-
*Diagnosis (ref* = *hematological)*					-		-	-	-		-	-		-	-		-
CNS tumor	-	-	-		-		-	-	-		-	-		-	-		-
Solid tumor	-	-	-		-		-	-	-		-	-		-	-		-
*Recurrence*	-	-	-		-		-	-	-		-	-		3.11	[0.95;10.22]		0.062
*Surgery*	-	-	-		-		-	-	-		-	-		-	-		-
*Chemotherapy*	-	-	-		-		-	-	-		-	-		-	-		-
*Radiotherapy*	0.52	[0.23;1.22]	0.135		-		-	-	-		-	-		-	-		-
**Psychosocial**																	
*PedsQL-YA Physical Functioning*	-	-	-		1.00		[0.97;1.03]	0.978	1.01		[0.98;1.04]	0.499		0.92	[0.96;1.01]		0.187
*PedsQL-YA Social Functioning*	-	-	-		0.96		[0.93;0.99]	0.005	0.96		[0.93;0.99]	0.012		1.00	[0.98;1.03]		0.937
*PedsQL-YA Work/School Functioning*	-	-	-		0.98		[0.96;1.01]	0.247	0.98		[0.95;1.01]	0.114		1.00	[0.97;1.02]		0.511
*HADS (sub)clinical anxiety (*≥ *8)*	3.61	[1.08;12.15]	0.038		1.54		[0.59;3.99]	0.378	2.44		[0.96;6.23]	0.061		1.76	[0.70;4.42]		0.231
*HADS (sub)clinical depression (*≥ *8)*	-	-	-		0.48		[0.12;1.98]	0.312	1.12		[0.24;5.15]	0.886		1.76	[0.42;7.36]		0.439
*CIS-20R severe fatigue (*≥ *35)*	-	-	-		2.31		[0.92;5.83]	0.075	1.42		[0.57;3.55]	0.455		1.00	[0.40;2.48]		1.0

- Not included in model; the following variables were not included in *any* model: Time since diagnosis, diagnosis, surgery (yes/no), chemotherapy (yes/no)

^a^YACCS with any need for support (%, *N*)

Abbreviations: *YACCS* Young Adult Childhood Cancer Survivor; *CNS* central nervous system; *PedsQL-YA* Pediatric Quality of Life–Young Adults; *HADS* Hospital Anxiety and Depression Scale; *CIS-20R* Individual Strength Revised

## Discussion

This study found that a large majority of YACCS report a need for support, in particular for information. This study added to the literature by specifically investigating the young adult subgroup of CCS and studying need for support in various domains and various support types. YACCS reported needs beyond information, with around one in 6 to one in 3 YACCS reporting a need for counseling across the domains.

Many YACCS reported a need for information, which was also demonstrated in previous studies [8, 11–13]. Information needs were the highest in the domains of physical consequences of childhood cancer and fertility which is in line with the results of previous studies [13, 32], and in the domain lifestyle and health risks. With information being reported as most needed on almost all domains, it seems that providing YACCS with age-appropriate information as early as possible should be a very high priority in survivorship care. In addition, from clinical practice, we know that medical information could impact survivors psychologically. Health care providers should be aware of this and be prepared to refer survivors for psychosocial support if necessary.

The psychosocial factors (sub)clinical anxiety and lower social functioning were identified as associates of higher overall need for support. More anxiety and poorer overall HRQOL were previously identified as predictors of more support needs [11, 14]. Through examining the various subdomains of HRQOL to gain a deeper understanding of which parts of HRQOL would influence support needs, we identified social function as the most relevant subdomain of overall HRQOL for needs. No medical characteristics were associated with the overall support needs.

We found different associated factors for support needs in specific domains. Support needs in certain domains (physical and social-emotional consequences of childhood cancer, relationships and sexuality, school and work, and future perspective) were mostly predicted by psychosocial factors, specifically lower social functioning and reporting (sub)clinical anxiety. Support needs in other domains (fertility, lifestyle and health after childhood cancer) were mostly predicted by sociodemographic characteristics such as female sex and higher educational attainment. The latter was not in line with previous studies. A study among survivors of AYA cancer found that those with lower educational attainment had more unmet needs [9] and a study of information needs in CCS found no effect of educational attainment [11]. The difference with earlier literature may be explained by the investigation of specific topics, like fertility and lifestyle and health after childhood cancer. While medical characteristics were not associated with needs in most domains in the present study, higher age at diagnosis and cancer recurrence were associated with need for support related to insurance and mortgages and relationships and sexuality.

Some specific results stood out. First of all, a need for support regarding fertility was strongly related to female sex and higher education, but not to any variables related to treatment that could cause infertility or any psychosocial variables. Need for support regarding sexuality, however, was significantly associated with lower social functioning and marginally associated with (sub)clinical anxiety. These results indicate that sexuality and fertility are subjects that are of interest to different subgroups of CCS and should both be discussed during survivorship care including the possibilities for support. Furthermore, looking at earlier literature about work and school performance of (YA)CCS [33, 34], it seems surprising that the need for support in this domain in the current study was the lowest among all domains (29.8%). It could be the case that problems relating to work and school are only pronounced in a small subset of the YACCS in this study. For example, central nervous system (CNS) tumor survivors were previously reported to be at an increased risk to experience problems related to school and work [33, 34].

### Implications

As young adulthood centers around the development of autonomy and identity [17], YACCS in particular should be empowered to take control of their own health. Currently, YACCS attendance of survivorship care is not optimal [8, 35], while there is evidence to suggest that they are vulnerable on both the physical and psychosocial levels [3, 5, 6, 20, 23, 25]. The suboptimal attendance is worrisome, because survivorship care is crucial to keep CCS as healthy as possible. CCS not attending survivorship care in (young) adulthood may be a result of a suboptimal transition from pediatric to adult care [36]. Making psychosocial survivorship care more tailored to the needs of CCS at all life stages, and during the vulnerable phase of young adulthood in particular, could help improve attendance. Insight into the needs of YACCS who did not attend survivorship care would be helpful. Unfortunately, the present study could not provide this insight because attendance of survivorship care was not assessed. Knowing what YACCS need is a first step to tailoring psychosocial survivorship care to their needs. Monitoring using patient reported outcomes in clinical practice could be useful to assess unmet needs and to monitor HRQOL as an indication of needs for which psychosocial support can be offered [37, 38].

This study stresses the need for adequate provision of information and information sources to YACCS during survivorship care. Having an accessible and age-appropriate information program could improve the participation of YACCS in their survivorship care [39, 40]. Looking at the results of the present study, information for YACCS should go beyond the physical consequences of childhood cancer and specific late effects, but also focus on emotional and social consequences. Besides providing information, health care providers should be encouraged to routinely discuss the possibilities for support, such as counseling, with YACCS in survivorship care [7]. YACCS in need of such psychosocial support have previously reported difficulties finding it [16]. Therefore, survivorship care centers should offer psychosocial support in addition to information provision directly to YACCS, or provide adequate referrals, usually to clinics in the network of care. To be of the best service to survivors, medical and psychosocial health care professionals need to work together multidisciplinary [16]. While doctors are responsible to provide patients with accurate medical information and advice, psychosocial care providers may help survivors attach a meaning to that information and cope with the impact this information has on them (e.g., counseling after news about infertility or a higher risk for subsequent tumors, or implementing lifestyle advice in daily life). YACCS could benefit from age-appropriate psychosocial interventions. Survivorship care clinics could specifically consider developing and offering interventions that can be delivered online, as the current events of the coronavirus disease 2019 (COVID-19) pandemic have forced us to consider more innovative ways to deliver psychosocial care away from hospitals or health care facilities. Online psychosocial care is especially compatible with survivorship care, because of the often low frequency of survivorship care clinic visits. Existing online interventions that could be used or adapted for YACCS include cognitive behavioral therapy-based group interventions such as Recapture Life-AYA and Op Koers Online [41, 42].

### Strengths and limitations

This study provides valuable insights into the specific needs of YACCS as a separate group from older CCS and survivors of AYA cancer. Looking at the few differences between responders and non-responders, we believe that stratifying the selection of YACCS was successful in obtaining a diverse sample.

Many previous studies on support needs in (YA)CCS were qualitative [16, 26], since needs are hard to quantify. Using a newly developed questionnaire provides the added value of quantification of YACCS’ needs in a novel way, specifically centering around the multiple types of support in domains that are relevant to YACCS and on several support types, including psychosocial needs and support. We studied needs in general rather than unmet needs, to reduce the influence of care that the YACCS receive at our institute and improve generalizability of our results to other institutes and countries.

Unfortunately, our analyses of associations in the specific support need domains were limited by the number of participants, so the results of the multivariate logistic regression analyses should be interpreted in an explorative way. Larger study samples are necessary to further investigate associations between support needs and sociodemographic and medical characteristics, as well as YACCS’ well-being. Larger studies could include variables that were not included in the present study, such as the presence and nature of late effects, or psychosocial factors such as coping.

## Conclusions

Most YACCS reported a need for support, in particular for information, especially regarding lifestyle and health risks after childhood cancer, physical consequences of childhood cancer, and fertility. Information provision including associated emotional consequences and support if necessary (psycho-education) should be at the base of survivorship care for YACCS, in order to meet their need for information as well as empower them to take control over their health during the crucial life phase of young adulthood. Health care providers should routinely discuss psychosocial well-being and consider possibilities for psychosocial support with YACCS and provide adequate referral when necessary.

## Data Availability

The datasets generated during and/or analyzed during the current study are available from the corresponding author on reasonable request.

## Appendix A: Support needs questionnaire (translated)

**In which domains do you need support?**

*In the first column there are domains in which support may be needed. In every other column, there is a type of support that you could need.*

*For each domain, please indicate which support types you need. You can tick multiple boxes for each domain. If you do not need any support in a domain, you can make this known by choosing the ‘none’ option.*

**Table:**

DOMAIN	CONCRETE INFORMATION	COUNSELING	PEER SUPPORT	OTHER	NONE
Physical consequences	○	○	○	○………………………	○
Social/emotional consequences	○	○	○	○………………………	○
Relationships and sexuality	○	○	○	○………………………	○
Fertility	○	○	○	○………………………	○
Lifestyle and health risks	○	○	○	○………………………	○
School/work	○	○	○	○………………………	○
Future perspective	○	○	○	○………………………	○
Mortgages and insurance	○	○	○	○………………………	○
Other areas………………………………	○	○	○	○………………………	○

## Code availability

N/A.
